# Biphasic anaphylactic reaction to blue dye during sentinel lymph node biopsy

**DOI:** 10.1186/1477-7819-6-79

**Published:** 2008-07-27

**Authors:** Margaret I Liang, William E Carson

**Affiliations:** 1 College of Medicine, The Ohio State University, Columbus, OH, USA; 2 Division of Surgical Oncology, The Ohio State University, Columbus, OH, USA

## Abstract

**Background:**

Lymphazurin 1% blue dye can cause a severe anaphylactic reaction in approximately 1–3% of patients.

**Case presentation:**

We describe a case of intraoperative anaphylaxis resulting from Lymphazurin 1% blue dye. A 48-year old woman undergoing a mastectomy with sentinel lymph node biopsy experienced a biphasic anaphylactic reaction with two episodes of hypotension at 15 minutes and 2 hours, respectively, after injection of the blue dye. The late phase was initially refractory to epinephrine.

**Conclusion:**

Early recognition, aggressive hemodynamic management, and prolonged monitoring are indicated in these patients to watch for a potential second phase anaphylactic reaction.

## Background

Sentinel lymph node biopsy (SLNB) has emerged as the standard procedure for staging of the axilla in patients with clinically node-negative breast cancer [1,2]. This procedure serves as an alternative to routine axillary lymph node dissection [3]. The sentinel lymph node is usually located by intraparenchymal injection of blue dye alone or in combination with intradermal administration of a radiolabeled colloid near the tumor site. In breast cancer, the combination of blue dye and radiotracer has been shown to markedly increase the sensitivity of SLNB [4-9]. Approximately 50% of the dye is weakly bound to serum albumin and is therefore selectively absorbed by lymphoid tissue [10,11]. The albumin-blue dye complex is picked up by regional afferent lymphatics, which causes lymphatic vessels and nodes to be identifiable by their bright blue color.

Lymphazurin 1%, also known as isosulfan blue, is the most commonly used blue dye in the United States. Severe allergic reaction and anaphylaxis have been observed in 1–3% of patients who are exposed to the dye during SLNB [12-14]. These allergic reactions can range from a mild allergic reaction characterized by urticaria and/or erythema, to anaphylaxis that is associated with hypotension, pulmonary edema, and/or cardiovascular collapse. Some investigators have advocated preoperative prophylaxis with steroids, diphenhydramine, and famotidine, with reported reductions in the severity but not the incidence of anaphylaxis [15]. The following case report describes an episode of severe anaphylaxis characterized by biphasic hypotension that occurred after intraparenchymal injection of Lymphazurin 1% for identification of the sentinel lymph node. The literature on the management of severe reactions is subsequently reviewed.

## Case presentation

A 48-year old woman was being treated for a malignant neoplasm of her left breast. The tumor was a moderately differentiated invasive ductal carcinoma (clinically T1 N0) that was discovered on physical examination and identified on subsequent mammogram. The various surgical options were discussed in depth with the patient, who decided not to undergo a breast conservation procedure or breast reconstruction. In addition, she elected to undergo a contralateral prophylactic mastectomy in order to address the approximate 0.5 – 1% per year risk of breast cancer in the unaffected breast. Therefore, her operative procedure was scheduled as a left modified radical mastectomy with SLNB and right prophylactic mastectomy. Her past medical history included migraine headaches and chronic sinus infections. Her past surgical history was significant for tonsillectomy, ankle surgery, dilation and curettage, and sinus surgery, all of which were performed under general anesthesia. The patient reported no drug, food, or other allergies. No previous perioperative anesthetic complications were reported by the patient.

On the morning of surgery, the patient was premedicated with 2 mg midazolam intravenously prior to the induction of anesthesia. General anesthesia was induced using fentanyl, propofol, and rocuronium, after which the patient was intubated in standard fashion. Anesthesia was maintained with nitrous oxide, oxygen, and isoflurane. The patient's left breast was then injected intraparenchymally with 5 mL of Lymphazurin 1% blue dye, which was followed by 5 minutes of light breast massage to mobilize the dye. Approximately 15 minutes later, after her chest wall had been prepped and draped, the patient experienced an acute episode consistent with cardiovascular collapse that was characterized by O_2 _desaturation and systolic blood pressures in the range of 30–40 mm Hg. The surgical procedure was halted (no incision had been made) and the patient was placed in the Trendelenburg position and given 100% oxygen. The possibility of a tension pneumothorax was eliminated by physical examination, which revealed robust breath sounds bilaterally. Intravenous fluids were administered (~2000 cc total) and 0.1 mg epinephrine (1:10 000) was given intravenously. Decadron (100 mg) and diphenhydramine (50 mg) were administered when the blood pressure failed to improve. She exhibited hives on her lower extremities bilaterally, but there was no blue discoloration to the skin. The patient was diagnosed with an anaphylactic reaction to the blue dye after these events. Her blood pressure stabilized and at that point she was placed on an epinephrine drip at 2 μg/min. The patient was then transferred to the surgical intensive care unit (SICU) for hemodynamic monitoring and ventilation management.

Approximately 2 hours after injection of the blue dye, the patient experienced a second hypotensive episode during which her systolic blood pressure dropped to 65 mm Hg. She received additional fluid resuscitation along with 50 mg benadryl, and the epinephrine drip was increased to 4 μg/min. The patient soon stabilized and the epinephrine was weaned the next day. She was discharged from the SICU 36 hours after admission. She was maintained on methylprednisolone, diphenhydramine, and famotidine, and given instructions on the use of an epinephrine pen and albuterol inhaler. The patient did well and exhibited no further allergic symptoms. She underwent the planned surgery 2 weeks later utilizing radioactive colloid alone to identify the sentinel lymph node. Two sentinel lymph nodes were identified that were negative for metastatic disease. Further examination of the lymph node specimens revealed a 3 mm metastatic focus in the first lymph node and a 6 mm metastatic focus in the second. The patient underwent completion lymphadenectomy 4 weeks later.

## Discussion

Lymphazurin 1% is the first dye of its type to be approved by the Food and Drug Administration for visualization of lymphatic tissues [16]. It is an aniline dye (2,5-disulfonated isomer of patent blue dye) with no known pharmacological action [17]. As described previously, approximately 50% of the total injection will weakly bind to serum proteins and will be selectively absorbed by the lymphatic vessels, allowing for identification of sentinel lymph nodes. Ninety percent of the blue dye is excreted via the biliary route, while 10% is excreted unchanged in the urine. Use of this dye is contraindicated in those individuals with a known hypersensitivity to triphenylmethane or related compounds (package insert). However, due to its widespread use outside medicine, including incorporation into textile dyes, cosmetics, hand lotions, household products, and paper, exposure and subsequent sensitization is likely to have occurred in a significant proportion of the population [18].

Anaphylactic reactions to the blue dye have been previously reported [10,11,16,19-21]. The incidence continues to increase due to the more frequent use of blue dye to delineate lymphatic spread of cancerous cells. In the operating room, the recognition of systemic anaphylaxis during general anesthesia depends almost entirely on the observation of clinical features in association with the temporal exposure to a foreign substance. Rather than laboratory tests, it is usually determined based upon clinical observations such as urticaria, erythema, respiratory complications, and/or cardiovascular collapse [22,23]. In most reported cases, patients develop symptoms within 30 minutes of blue dye injection. The patient in this report experienced a rapid decrease in blood pressure with a systolic pressure declining to 40 mm Hg within minutes after the injection of 5 mL of Lymphazurin 1%. Numerous cases have been reported in which patients display a systemic urticarial rash with blue coloration from the blue dye along with the anaphylactic reaction, but these events may also occur independently of each other [24]. The present case differs in that the patient exhibited hives bilaterally on her lower extremities (these were initially hidden by sequential compression devices), but there was no blue skin discoloration.

Although rare, anaphylactic shock after administration of blue dye for SLNB is a potentially lethal situation. Early recognition as well as aggressive hemodynamic management of these reactions can dramatically reduce morbidity and mortality [25]. In general, initial treatment modalities should be targeted toward blood pressure management and airway support. All anesthetic agents should be immediately discontinued and 100% oxygen and rapid intravenous infusion of crystalloids should be promptly instituted. For initial pharmacologic management of acute anaphylaxis, epinephrine should be administered immediately [26]. Studies have shown that a delay in the administration of epinephrine, the use of an inadequate amount of epinephrine during the first phase, or a requirement of large doses of epinephrine to ameliorate the initial response might predispose to a biphasic response [26-29]. While epinephrine has not been shown to consistently prevent the second reaction, it remains the treatment of choice for anaphylactic reactions. Intravenous epinephrine (1:10 000) is typically only administered in severe hypotensive shock, as in this case, because of the potential for inducing tachyarrhythmias. Intravenous antihistamines (H1 and H2 blockers) should be considered next if the reaction persists because they can reverse the effects of systemic histamine release and thereby alter vascular permeability and systemic hemodynamics [22,23,30,31]. Corticosteroids can also be given concurrently to minimize or prevent the second phase reaction of anaphylaxis, as this has been demonstrated to be beneficial in some individuals [32-35]. There is no consensus as to whether the administration of corticosteroids affects the incidence of a late reaction. Of note, there have also been several documented cases of patients who received corticosteroid therapy and still went on to experience biphasic reactions [35-37].

We report a case in which a patient experienced a biphasic anaphylactic reaction to Lymphazurin 1% blue dye during SLNB. Interestingly, the second hypotensive episode occurred within 1 hour of successful management of the first phase. Corticosteroids were administered in this case, but did not prevent a biphasic reaction in our patient. Moreover, this late phase reaction was initially refractory to epinephrine as the patient was already on an epinephrine drip when the late phase of her anaphylactic reaction occurred. Biphasic anaphylactic reactions in which late recurrences of hypotension occur several hours after the acute episode have been previously reported. Albo *et al. *described two patients who experienced biphasic anaphylactic reactions. In both cases, the first episode of anaphylaxis was managed by administration of crystalloid, phenylephrine, epinephrine, hydrocortisone, and diphenhydramine. Both patients had a second episode of anaphylaxis during postoperative monitoring (6 hours and 8 hours after surgery, respectively). The severity of their second reactions was not reported, but they were both treated successfully with an epinephrine bolus and infusion of 1000 cc of crystalloid. Beenen *et al. *described a patient who experienced a second period of hypotension [38]. After injection of the blue dye, a severe decline in the blood pressure was witnessed. This initial anaphylactic reaction was controlled with ephedrine, tavegyl, and prednisone. The patient recovered and a SLNB was performed, but when the surgeon wanted to continue with resection of the left breast, a second period of hypotension occurred. No urticaria was observed and this subsequent episode was successfully treated with epinephrine. These biphasic anaphylactic reactions could be caused by delayed systemic release of antigen stores from the tissue compartment back into the circulation once the circulating levels of blue dye begin to undergo clearance from the bloodstream. Another suggested mechanism for the second phase of anaphylaxis is due to the recruitment of late inflammatory mediators, including prostaglandins, leukotrienes, and nitric oxide.

Thus, patients who exhibit any sort of hemodynamic instability should not go on to have further surgery at that same setting. Also, a longer period of observation of up to 24 hours is indicated in patients who experience an anaphylactic reaction to blue dye before the episode should be considered fully resolved [13,18,39].

## Conclusion

As the use of Lymphazurin 1% for SLNB in the staging and management of breast cancer becomes increasingly common, we will likely see an accompanying rise in the incidence of anaphylactic reactions to blue dye. It is essential that the personnel involved in the performance of those procedures involving blue dye for lymphatic visualization are aware of and prepared to recognize and treat anaphylaxis. Most importantly, this case report highlights the need for extended observation and careful monitoring of these patients for the possibility of biphasic anaphylactic reaction that may occur hours after the apparent resolution of an acute episode of anaphylaxis to blue dye.

## Competing interests

The authors declare that they have no competing interests.

## Authors' contributions

MIL wrote and edited the manuscript. WEC was a surgeon involved in the case who developed and oversaw the project. He also helped to write the manuscript. All authors read and approved the final manuscript.
